# Proceedings of the 2010 MidSouth Computational Biology and Bioinformatics Society (MCBIOS) Conference

**DOI:** 10.1186/1471-2105-11-S6-S1

**Published:** 2010-10-07

**Authors:** Jonathan D Wren, Doris M Kupfer, Edward J Perkins, Susan Bridges, Daniel Berleant

**Affiliations:** 1 Arthritis and Immunology Research Program, Oklahoma Medical Research Foundation; 825 N.E. 13th Street, Oklahoma City, OK 73104-5005, USA; 2 Aerospace Medical Institute, Federal Aviation Administration, Oklahoma City, OK 73169, USA; 3 US Army Engineer Research and Development Center, 3909 Halls Ferry Road, Vicksburg, MS 39180, USA; 4 Department of Computer Science and Engineering, Mississippi State University, Box 9637, Mississippi State, MS 3976, USA; 5 Department of Information Science, University of Arkansas at Little Rock, Little Rock, AR 72204, USA

## Introduction

The seventh annual Midsouth Computational Biology and Bioinformatics (MCBIOS) conference took place February 19 and 20, 2010 at Arkansas State University in Jonesboro, AR, presided over by Daniel Berleant, this year’s President of MCBIOS.  Keynote speakers were Elaine Ostrander of NIH, renowned for her work on dog genomics, Clayton Naeve, CIO of St. Jude Children's Research Hospital, and Robert Cottingham, Leader of the Computational Biology and Bioinformatics Group, Oak Ridge National Laboratory. The record-breaking attendance exceeded 200 participants which necessitated parallel talk sessions for the first time. Student oral presentation award winners were Heidi Pagan (first place) of Mississippi State University (MSU), Juliet Tang (second place) of MSU, and Aleksandra Markovets (third) of Mississippi Valley State University. In addition, a record number of posters were also presented. Due to the number of posters, student poster awards were given in two categories, Biological Focus and Computational Focus. Winners were N. Platt and V. Chaitankar (1st place), G. Cooper and L. Pillai (2nd), and M. Ammari and C. Gearheart (3rd). Reflecting the integration of these foci in the field, winners in one category frequently scored high in the other category as well. MCBIOS is also pleased to have this year acquired legal status as a non-profit organization.

## Proceedings summary

A total of twenty eight papers describing research presented at the 2010 conference were accepted for publication in these proceedings out of a total of forty three papers submitted for consideration (~65%). The number of papers submitted for consideration was the largest submitted since the inception of MCBIOS with a 34% increase over last year’s proceedings [1-19], and the highest number of papers submitted to date, reflecting a healthy growth in conference participation. All papers were peer-reviewed by at least two reviewers. Our goal for inclusion of papers was to be inclusive, yet rigorous in the peer-review process such that accepted papers are both high quality and reflective of the work presented at the conference. Papers generally fell into five categories: 

## Genomic analysis

Bioinformatics was largely born as a field through the need to analyze and understand sequence data. Partly as a result of ultra-high-throughput sequencing technology, the field has seen a strong resurgence recently with new computational methods being developed and applied to downstream applications such as the identification of human mutations [20-25] as well as meta-genomics and whole genome sequencing [26-31]. In these proceedings, several new methods and applications are reported. Hong Fang, *et al*. describe an expansion of the FDA-developed, ArrayTrack^TM^ genomics tool, into a platform that supports microbial data to enable the rapid detection of food borne pathogenic bacteria during outbreak scenarios [32].  Xu *et al*. describe new SNP (single nucleotide polymorphism) and QTL (quantitative trait locus) libraries for the ArrayTrack system which provide users with extensive data including links to other resources, and motivated in part by the need to augment gene association study results with biological context [33]. Christopher Bottoms *et al.* report on the new web-based tool, IView, which graphically displays and makes searchable introgression data by marker name, chromosome number, or map position [34].

Quest and colleagues working in the Cottingham laboratory address the problem of brittle genome annotation systems with an OWL-based approach that provides several significant advantages [35]. Work reported by Markovets and colleagues on identifying promoter regions showed that a new neural network-based algorithm outperformed the method of stress-induced duplex destabilization [36]. Smits and Ouverney present a software system that assists users in analyzing sequence data to find phylogenetic trees, while avoiding pitfalls associated with this process as commonly practiced [37].

An examination of substitution rates over evolutionary time by Ulrich Melcher compared viral and non-viral sequences to reveal that the rate of nucleotide interchanges in plant viruses is not constant, impacting phylogeny studies.  The evidence suggests that different evolutionary rules may apply to recent divergences than to those linked to distant speciation events [38].  

An in depth analysis of the two forms of Bovine Viral Diarrhea, a worldwide cattle disease, was completed by Mais Ammari *et al*. using a proteomics approach to evaluate the host protein expression differences. Two gene sets were found, each specific to one disease form which showed significant functional differences [39].  Arun Rawat *et al*. address the need for additional genomic information for Northern bobwhite, an avian species critical to ecosystem maintenance. Using next generation sequencing of cDNAs from a multi-tissue library, they generated ESTs which were pipelined into a unigene database and made publicly available at a searchable website [40].  

Cyriac Kandoth *et al *[41] mined the Arabidopsis thaliana genome using a new method to scan genome sequences for partially symmetric inverted repeats that might represent miRNA precursors. Chao Di *et al *[42] carried out a comparative genome analysis of the Prohibitin gene, important functions from diseases to development, finding that the gene family is conserved across different kingdoms falling into five different clades and indicating that different duplication events were involved for gene family expansion, especially the segmental duplications in *Arabidopsis*. The conserved gene evolution indicated that the study in the model species can be translational to human disease studies. Rowena Kelly *et al *[43] have constructed a database with a web-based interface that integrates large datasets on *Aspergillus flavus* resistance in Maize such as gene expression, proteomic, QTL genetic mapping studies and sequence data from the literature to help identify and select candidate *A. flavus* resistance genes.

### 3D/structural analysis

Biological information may be encoded in a one-dimensional sequence, but its manifestation takes place in three-dimensional space in the form of proteins, cells and tissues among other structures. Computational analysis and prediction of these structures is challenging, to say the least, and an active area of recent bioinformatics interest [44-48]. Computational simulations are critical for testing proposed solutions, as well as in rationally engineering more specific binding/activity for drug candidates.

To better understand and improve of the creation of anti-inflammatory drugs through inhibition of lipoxygenase, an essential enzyme in the inflammatory pathway, Shuju Bai *et al.* report on a method for modeling interactions between 8R-lipoxygenase and its substrate [49]. A mixed reality surgical simulator with a VICON motion tracking system, developed for the rasping procedure in artificial cervical disc replacement surgeries is described by Tansel Halic *et al.*, which can serve as an important training and practice tool for surgeons [50].  The potential of using hydrogen deuterium exchange (HDX) mass spectrometry for analyzing enzymes that degrade cell walls is explored by Uzuner *et al.*[51].  They report that the HDX mass spectrometry can be a powerful tool for exploring the molecular mechanisms of enzyme functions.  

Sinan Kockara *et al *[52] and Mutlu Mete *et al *[53] examined ways to automatically quantitate and define the extent of malignant melanomas on skin while offering speed and consistency in detection of the lesions borders, elements especially useful in areas without dermatologists. Kockara *et al * compared density based clustering and Fuzzy C-Means clustering algorithms for border detection in dermoscopy images finding that density based clustering performed best with a low border error, high precision and recall; however, the Fuzzy C-Means clustering algorithm had poor performance especially in border detection.  Mete *et al * proposed two new methods, boundary driven density based clustering based algorithms which performed better delineation with noisy images and an active contour model that gave the best detection with optimum parameters. Denise Koessler *et al *[54] developed a tool to predict secondary RNA structures based on the novel approach of using graph-theoretic values as input for a neural network and computes the probability of secondary RNA structure.

## Systems biology

There are many areas within biology that are amenable to computational analysis, each of which is usually approached separately. Somewhat recently, the term “systems biology” has emerged to describe interconnected analyses, particularly those that help reduce complexity in these systems, as defined by the number of interconnections between component parts, into a more functional understanding (e.g., input and output). The term “systems biology” is often used broadly and not always consistently, but is an emergent area of high interest in bioinformatics [55-57]. 

A novel method for identifying subnetwork markers in a human protein-protein interaction network is described by Junjie Su *et al.*[58].  They report the identification of subnetwork markers that improve upon current gene-based and pathway-based markers in their discriminative power, reproducibility and classification. Elina Tjioe *et al.* report on the development of a Web-based gene-discovery bioinformatics tool, FAUN (Feature Annotation Using Nonnegative matrix factorization), which identifies implied gene relationships from biomedical literature, enabling hypotheses on functional characterization [59].  

 A paper by Vijender Chaitankar, *et al*., explores the important problem of inferring gene regulatory networks from time sliced microarray data. They claim that mutual information (MI) based approaches have inherent limits to their capabilities in this context which they were able to improve upon with algorithms based on modifications to the mutual information metric [60].

Kumar and Nanduri present a downloadable database, HPIDB, which serves as a resource of host-pathogen protein-protein interactions integrated non-redundantly from public databases. The authors report on the flexibility of the database for querying and an output format which allows portage to downstream applications [61].

## Microarray studies

Microarrays have long been a subject of bioinformatics analysis not only for better understanding transcription, a process affected by both genetic and epigenetic factors [62,63], but also as a model for large-scale analysis of genetic behavior. 

A new non-stationary Dynamic Bayesian Network with a flexible lag choosing mechanism that detects potential genetic regulators and has improved structure prediction is reported by Yi Jia and Jun Huan [64].

Zhining Wen *et al*. address two interesting QC questions of whether expired Affymetrix GeneChip microarrays, an expensive resource, are still useful up to four years after expiration and if RNA stored at -80°C for the same period was suitable as template source.  In spite of a decrease in sensitivity, the authors found that these arrays generated data consistent with that from unexpired arrays and report favorably on the stability of the RNA [65].

## Miscellaneous

There are a few other papers published in the proceedings but, for space, are not summarized here: [66-69].

## Future meetings

MCBIOS 2011 will be held in College Station, Texas.  The 2010-2011 President of MCBIOS is Ulisses Braga-Neto of the University of Texas A&M, and Susan Bridges of Mississippi State University is now the President-elect. MCBIOS is a regional affiliate of the International Society for Computational Biology (http://www.ISCB.org). For information regarding MCBIOS and our future meetings, see http://www.MCBIOS.org.

## Competing interests

The authors have no competing interests to declare.

## Author contributions

All authors served as editors for these proceedings, with JDW serving as Senior Editor. All authors helped write this editorial. 
